# Preparation and Properties of High-Toughness AlMgB_14_ Material

**DOI:** 10.3390/nano15100764

**Published:** 2025-05-19

**Authors:** Tianxing Sun, Zhaohua Luo, Yusen Duan, Jingxian Zhang

**Affiliations:** 1 University of Chinese Academy of Sciences, Beijing 100049, China; suntianxing22@mails.ucas.ac.cn; 2 The State Key Lab of High Performance Ceramics and Superfine Microstructure, Shanghai Institute of Ceramics, Chinese Academy of Sciences, Shanghai 200050, China; luozhaohua@mail.sic.ac.cn (Z.L.); duanyusen@mail.sic.ac.cn (Y.D.)

**Keywords:** AlMgB_14_, ceramic composites, solid solution, fracture toughness

## Abstract

This study employed a composite method using TiB_2_-HfC dual-component additive to prepare AlMgB_14_ ceramic composite material. The morphology and phase composition of the AlMgB_14_ ceramic powder were characterized using scanning electron microscopy (SEM) and an X-ray diffractometer (XRD). The phase evolution, microstructure, and mechanical properties of the sintered composite were investigated. The experimental results indicate that the AlMgB_14_-based composite sintered at 1450 °C exhibited excellent comprehensive properties, with a Vickers hardness of 25.3 GPa, a fracture toughness of 6.9 MPa·m^1/2^, a bending strength of 615 MPa, and a density of 3.22 g/cm^3^. Additionally, a solid solution second phase was observed in the AlMgB_14_ material. Through a dual-component synergistic composite strategy, this study enhanced the toughness of AlMgB_14_ material without significantly compromising other properties, providing a new design approach for the development of low-cost, high-performance AlMgB_14_-based composites.

## 1. Introduction

Hard materials, as the core basic materials of modern manufacturing, have always been a research hotspot in the field of materials science for their performance optimization. Traditional hard materials such as alumina ceramics have drawbacks such as insufficient hardness and high density [1], and they also face common problems such as low toughness and high brittleness. On the other hand, ultra-hard materials like diamond are limited by stringent synthesis conditions and brittleness as well. Additionally, cracking caused by insufficient toughness also restricts their application. Therefore, the development of a new high-performance, hard material system has significant engineering application value.

In 1970, Matkovich et al. [2] first discovered a ternary boride impurity phase with a stoichiometry close to AlMgB_14_ during the synthesis of AlB_12_, and the density of AlMgB_14_ is about 2.60 g/cm^3^. Higashi et al. [3] analyzed its crystal structure and confirmed that this phase has a three-dimensional network structure built from B_12_ icosahedra. Cook’s team [1] first proposed, in 2000, that AlMgB_14_ could be a candidate material for a new type of superhard material system. The composites prepared by their team had a hardness of over 40 GPa and a density of only 2.70 g/cm^3^. The excellent comprehensive properties of AlMgB_14_ material and its low-symmetry structure, different from traditional hard materials [3,4], have sparked widespread academic research on AlMgB_14_-based materials. Currently, the preparation of AlMgB_14_ mainly employs processes such as hot pressing sintering (HPS) [1,5,6,7,8,9,10,11,12,13,14,15,16,17,18,19], spark plasma sintering (SPS) [20,21,22,23,24,25,26,27], field-activated and pressure-assisted sintering (FAPAS) [28,29,30,31,32,33], and self-propagating high-temperature synthesis (SHS) [34,35,36]. Compared with other preparation methods, hot pressing sintering can conveniently set the thermal regime of the heat treatment process and can prepare samples of various sizes and shapes. Due to the ease of forming various intermediate phases during the synthesis process, a stepwise heat treatment strategy can be adopted [37,38,39], initially obtaining a precursor through calcination at lower temperatures and then achieving the synthesis and densification of the target product through high-temperature sintering.

At present, extensive discussions have been held on the hardness enhancement of AlMgB_14_-based ceramic materials, but the improvement of AlMgB_14_ ceramic toughness has received little attention. The toughness of the reported AlMgB_14_ ceramic materials is mostly at a low level, and the fracture toughness of AlMgB_14_-based materials prepared by conventional modification schemes is mostly in the range of 3–4 MPa·m^1/2^ [7,8,13,23,29,31,33]. The lack of crack propagation resistance can lead to brittle fracture under dynamic load conditions such as cutting, processing, and impact protection, which may cause failure during the service of equipment components. Some researchers have noticed the issue of toughness enhancement in AlMgB_14_ materials and have attempted to regulate material properties by introducing metal additives [6,19]. This method has successfully increased the toughness of the material to about 10.7 MPa·m^1/2^; however, the use of metal additives has resulted in a significant decrease in other properties such as hardness and strength of the material. Therefore, how to improve the toughness of the material without compromising other properties has become an urgent problem to be solved.

Given that the existing research on AlMgB_14_ materials has mainly focused on the introduction of single additives [1,8,9,11,12,13,26,32,33,34,36,38,39], such as the elemental or compound forms of Ti or Si, reports on a synergistic composite method with multiple additives are scarce. This study innovatively employs a dual-component synergistic reinforcement strategy utilizing TiB_2_-HfC to prepare AlMgB_14_-based composite materials through a two-step heat treatment process. Except for the high-temperature heat treatment stage, the rest of the processes are completed in an air environment, significantly reducing the difficulty and cost of preparation. Through an in-depth discussion of the phase composition, microstructure of the composite materials, and the mechanism of their influence on mechanical properties, this study provides a new approach for the development of high-performance AlMgB_14_-based ceramics.

## 2. Materials and Methods

### 2.1. Materials

**Figure 1:**
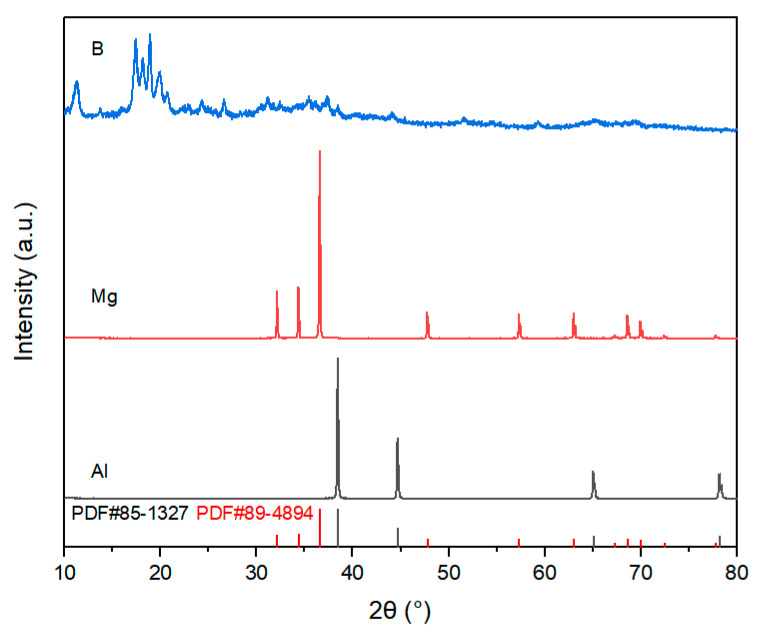
XRD patterns of raw materials.

**Figure 2:**
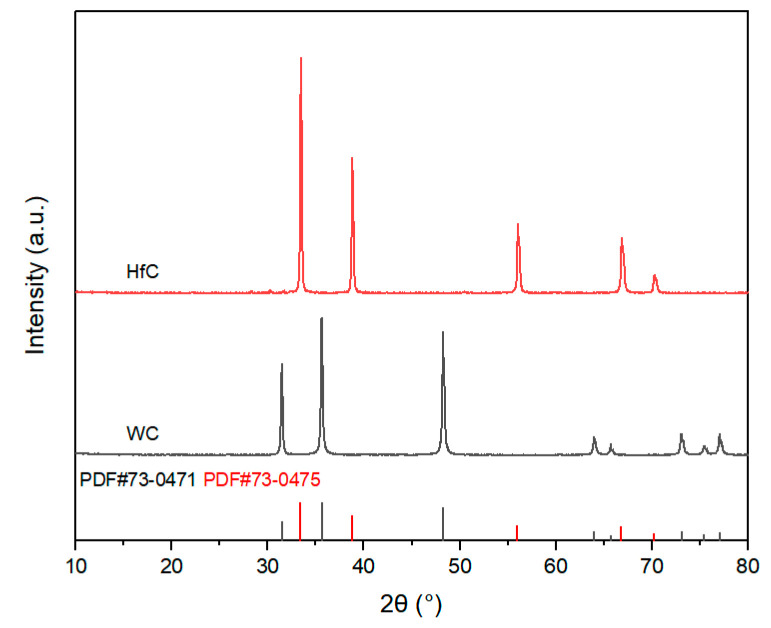
XRD patterns of additives.

The preparation process of this study used commercially available high-purity powders as raw materials. Aluminum (Al) powder, magnesium (Mg) powder, and amorphous boron (B) powder were used as raw materials for synthesizing AlMgB_14_ ceramics. TiB_2_ powder and HfC powder were used as additives. For more detailed information regarding the manufacturers and specifications of the materials used, please refer to Table 1. The XRD patterns of the raw materials and the additives can be seen in Figure 1 and Figure 2.

### 2.2. Preparation of AlMgB_14_ Ceramic Powder

In the powder preparation stage, Al, Mg, and B powders were mixed in a 1:1:14 molar ratio using a planetary ball mill with ethanol as the medium, silicon nitride balls were utilized. After ball milling, the evenly mixed slurry was placed in the oven to dry, then it was ground into powder with a mortar and sieved through a 60-mesh sieve. The sieved powder is pressed using a steel mold to form a block that is easy to place into the furnace. The block is then placed into the furnace and heated at a rate of 10 °C per minute to a high temperature point, with a holding time of 5 h. The six powders are calcined in the temperature range of 950 °C to 1200 °C, with an increment of 50 °C. During the calcination process, argon gas is injected into the furnace for protection, and the block is cooled to room temperature with the furnace after heating. After heat treatment, the block is ground into fine powder and sieved through a 150-mesh sieve for future use.

### 2.3. Sintering of AlMgB_14_ Ceramic

After sieving, the ceramic powder was subjected to the second round of ball milling, during which the additive was added at a concentration of 30 wt.%. After ball milling, the slurry was dried, followed by grinding and sieving through a 150-mesh sieve. The powder to be sintered was pressed into a block with a cross-section size of 35 mm × 30 mm using a steel mold. The ceramic green body was placed in the graphite die, sintered in an argon atmosphere under 40 MPa uniaxial pressure at 1450 °C with a holding time of 3 h, and then cooled to room temperature with the furnace. Dense ceramic bulk material with a dark gray appearance was obtained by sintering. To facilitate the construction of the control groups, the bulk ceramic materials were divided into three types of samples. The first type of sample used the original powder without adding other substances for sintering. The original powder group was divided into two: one that underwent a second round of ball milling after the first heat treatment step and one that did not. The second type of samples utilized powder with a single additive (TiB_2_ or HfC) added for sintering. The third type of sample used powder with two additives (TiB_2_ + HfC in a 1:1 mass ratio) introduced for sintering.

### 2.4. Characterization

Phase composition analysis of the ceramic powders and bulks was conducted using an X-ray diffractometer (D8 DISCOVER DAVINCI, Stuttgart, Germany) with CuKα radiation and based on the «Powder Diffraction File» database. The scanning parameter was: 10–80° 2θ diffraction angle range with 10°/min scanning speed; the scanning step was 0.02°.

After sintering, the ceramic blocks were processed into standard specimens of size 3 mm × 4 mm × 35 mm by wire cutting. After ultrasonic cleaning with anhydrous ethanol, the three-point bending test was conducted using a universal testing machine (Instron-5566, Buckinghamshire, UK) with a moving crosshead speed of 0.5 mm/min and the span was 30 mm. The fractured samples obtained during the test were subjected to surface polishing. The polished surface of bulk material was finished with polycrystalline diamond suspension to a sub-micron surface for hardness testing using a Vikers hardness tester (Shimadzu HMV-G, Kyoto, Japan) with a load of 1 kg and a dwell time of 15 s. The fracture toughness (K_IC_) was calculated based on the indentation cracks. The calculation method can be seen in Formula (1) [40,41]:

(1) $$K_{\mathrm{IC}}=P{\left(\pi \left(\frac{C_{1}+C_{2}}{4}\right)\right)}^{-\;\frac{3}{2}}{\left(\mathrm{tg}\beta \right)}^{-1}$$

In the formula, P represents the indentation load, C_1_ and C_2_ represent the measured diagonal crack lengths, tg means tangent, and β is an angular constant (68°).

Microstructure observation and elemental distribution analysis were completed using a scanning electron microscope (Verios G4, Hillsboro, OR, USA/SUPRA 55 SAPPHIRE, Oberkochen, Germany) combined with energy dispersive spectroscopy (EDS). Powders were dispersed with ethanol, and the bulk materials were coated with Cr. The bulk materials were the polished bulks that had undergone hardness testing.

Additionally, density testing employed the Archimedes method, with 6 fractured samples tested for each group, using 25 °C deionized water as the medium.

## 3. Results and Discussion

### 3.1. Phase Composition and Micromorphology of AlMgB_14_ Ceramic Powder

**Figure 3:**
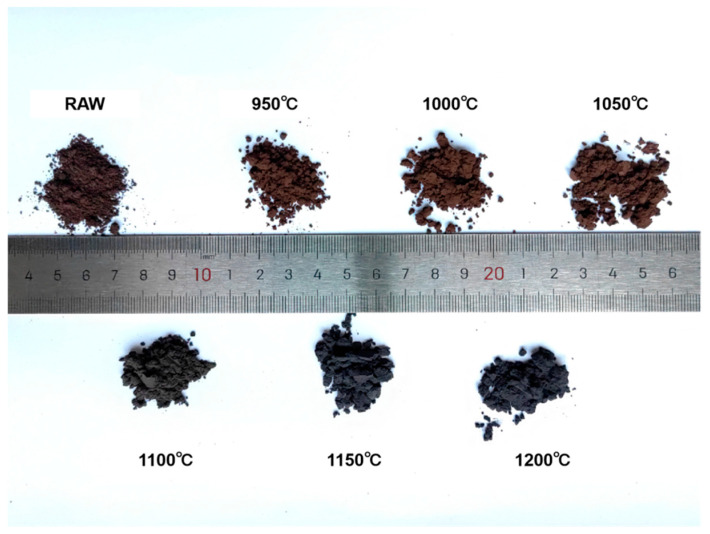
Photo of powders treated under different temperatures (including RAW).

The samples were heat treated at different temperatures from 950 °C to 1200 °C in steps of 50 °C. The macro-morphology of the powder exhibits significant temperature dependence (see Figure 3). In the range of 950–1050 °C, the powder maintains the loamy texture of the raw mixed powder (referred to as RAW), with its brown characteristics attributed to the mixture of unreacted amorphous boron and intermediate products of the Al-Mg-B system. When the temperature rises to 1100 °C, the color transitions to a brownish-gray transitional state. Powders treated at temperatures of 1150 °C and above completely turn gray and exhibit a sandy granular morphology.

**Figure 4:**
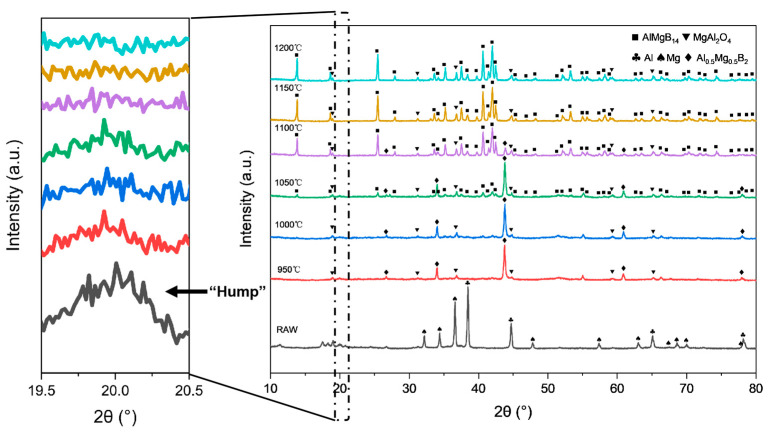
XRD patterns of powders obtained from heat treatment at different temperatures (including RAW).

Based on the XRD analysis results (see Figure 4 and Table 2), compared with the XRD patterns of the raw materials (see Figure 1). The mixed raw material powder without heat treatment consists of pure Al, pure Mg, and amorphous B, with the ball milling process only achieving mechanical mixing without inducing chemical reactions. After heat treatment at 950–1050 °C, the main phase of the powder evolves into Al_0.5_Mg_0.5_B_2_ and MgAl_2_O_4_, where the sample heat-treated at 1050 °C shows weak diffraction peaks of the AlMgB_14_ phase for the first time. When the temperature rises to 1100 °C, the diffraction peak intensity of the AlMgB_14_ phase significantly increases and the peaks become sharp, while the peak intensity of the Al_0.5_Mg_0.5_B_2_ phase relatively weakens, forming a multiphase structure where AlMgB_14_, MgAl_2_O_4_, and Al_0.5_Mg_0.5_B_2_ coexist. Under the heat treatment condition at 1150 °C, the diffraction peaks of the Al_0.5_Mg_0.5_B_2_ phase disappear, indicating that this intermediate phase has transformed into the AlMgB_14_ phase. At this point, the material system is composed of the AlMgB_14_ main phase and the MgAl_2_O_4_ oxide phase. When the temperature is further increased to 1200 °C, the XRD pattern characteristics are basically consistent with those of the sample obtained at 1150 °C, and no new phase generation or significant phase ratio changes are observed, confirming that the AlMgB_14_ phase has reached a thermodynamically stable state at temperatures above 1150 °C.

**Figure 5:**
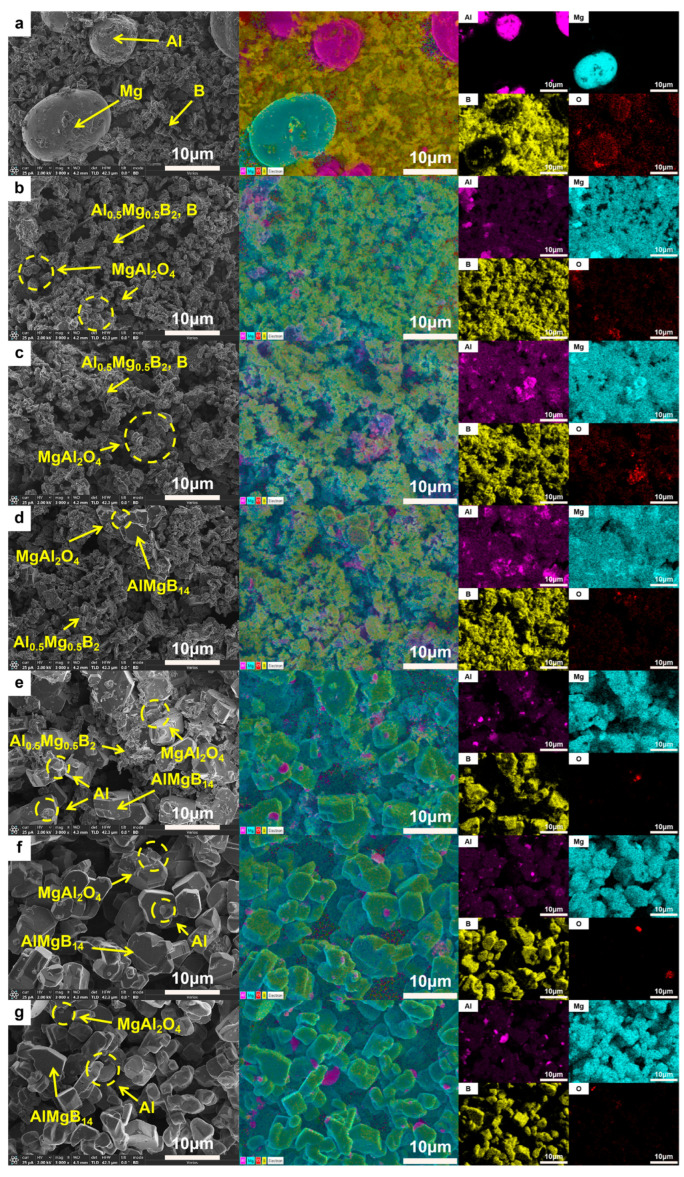
SEM images and EDS result of different powders: (**a**) RAW; (**b**) 950 °C; (**c**) 1000 °C; (**d**) 1050 °C; (**e**) 1100 °C; (**f**) 1150 °C; (**g**) 1200 °C.

The morphology of the ceramic powders was observed using a scanning electron microscope. The SEM images of each powder are shown in Figure 5. The raw material powder was composed of mechanically mixed elemental powders, and within the powder, fine amorphous B and spherical Al and Mg were observed. After heat treatment at 950 °C, the spherical metal particles were no longer visible, and the powders exhibited a morphology similar to that of the raw amorphous B powder. Additionally, sheet-like or blocky crystals with relatively regular appearances could be observed. Combining the results of XRD analysis, it can be inferred that these were crystals of the Al_0.5_Mg_0.5_B_2_ phase. Furthermore, a small amount of Al-Mg alloy structure formed by the melting and subsequent solidification of metals that did not react with B could be observed, with O element enriched in these areas. When the heat treatment temperature was increased to 1000 °C, a significant increase in regular crystal materials could be observed, with grain sizes reaching about 5 μm. Additionally, a reduction trend in the amorphous material was evident, as indicated by the weakening “hump” in the XRD patterns with the rise in treatment temperature. When the heat treatment temperature was further increased to 1050 °C, large blocky crystals with clear edges began to appear in the powder. The size of these crystals could reach 10 μm. Combining the diffraction peaks of the AlMgB_14_ phase that began to appear in the XRD pattern and the EDS results (see Figure 5), it can be inferred that these crystals are AlMgB_14_. When the heat treatment temperature reached 1100 °C, the morphology of the powder underwent significant changes compared to that obtained at lower heat treatment temperatures. The amorphous material in the powder was greatly reduced, XRD pattern shows that the “hump” is almost invisible, and there were a large number of regularly shaped blocky crystals in the powder. At this point, the relative intensity of the diffraction peaks of the Al_0.5_Mg_0.5_B_2_ phase (visible in the 2θ = 43–45° region) significantly decreased, while the relative intensity of the diffraction peaks of the AlMgB_14_ phase (visible in the 2θ = 39–43° region) significantly increased. It can be inferred that 1100 °C is a critical temperature point for a large transformation of the Al_0.5_Mg_0.5_B_2_ phase to the AlMgB_14_ phase. When the temperature reached 1150 °C, the XRD pattern shows that the “hump” had disappeared, and no amorphous phase could be observed in the powder. The raw material was almost completely converted into the target product AlMgB_14_, and MgAl_2_O_4_ oxide impurities and a very small amount of Al could be detected. The presence of Al can be attributed to the release of Al element during the conversion of low borides to AlMgB_14_ [42]. The situation of the powder treated at 1200 °C was similar to that treated at 1150 °C, indicating that AlMgB_14_ reached a thermodynamic stable state at 1150 °C.

Based on the systematic characterization results of ceramic powders obtained from different heat treatment temperatures, this study reveals the possible reaction route of AlMgB_14_, which can be specifically divided into the following evolution stages:

Al + Mg + 4B = 2Al_0.5_Mg_0.5_B_2_, (2)

14Al_0.5_Mg_0.5_B_2_ = 2AlMgB_14_ + 5Al + 5Mg, (3)

The evolutionary patterns inferred by this study are highly consistent with the reaction route proposed by Roberts et al. [42]. It also, to some extent, explains the temperature-dependent morphological evolution of Al-enriched regions, the phenomenon primarily attributed to the behavioral characteristics of metal elements during the reaction process, where some metal elements are released during the transition from low borides to high borides (AlMgB_14_). Metallic Mg, due to its relatively high vapor pressure [43,44,45], is more prone to loss under high-temperature conditions, making it difficult to observe in the final product; due to the relatively low vapor pressure of Al, it is possible for Al to form a melt, subsequently maintaining a near-spherical shape during the cooling process within the powder.

XRD and EDS analyses indicate that the treated powder contained MgAl_2_O_4_ impurities. Given that the entire heat treatment process was carried out under argon protection and did not involve oxygen, the oxide impurities should originate from the oxidation reactions during the ball milling stage of the powder. Possible side reactions are as follows [25,46,47,48,49]:

Al_2_O_3_ + MgO = MgAl_2_O_4_, (4)

It may also come from the reaction between metal and B_2_O_3_ [24]:

6Al + 3Mg + 4B_2_O_3_ = 3MgAl_2_O_4_ + 8B, (5)

Al_12_Mg_17_ + 8B_2_O_3_ = 6MgAl_2_O_4_ + 16B + 11Mg, (6)

In summary, 1050 °C and 1100 °C correspond to two critical phase transition temperatures for the synthesis of AlMgB_14_. Under the molar ratio of A:Mg:B = 1:1:14, the reconstruction reaction from low borides towards high borides begins at 1050 °C, leading to the formation of the AlMgB_14_ phase. When the temperature rises to 1100 °C, the powder achieves rapid conversion of the intermediate product to the AlMgB_14_.

### 3.2. Phase Composition and Microstructure of Sintered AlMgB_14_ Ceramic Materials

Considering the decomposition issue of AlMgB_14_ [24,39], the powder prepared at 950 °C was used for the sintering of bulk materials. The ceramic material obtained by sintering is a dark gray block. The cross-section has a gray metallic luster, and a bright mirror surface can be obtained after polishing. The phase composition of sintered ceramic materials can be seen in Table 3.

#### 3.2.1. Phase Composition and Microstructure of AlMgB_14_ Ceramic Without Modification

**Figure 6:**
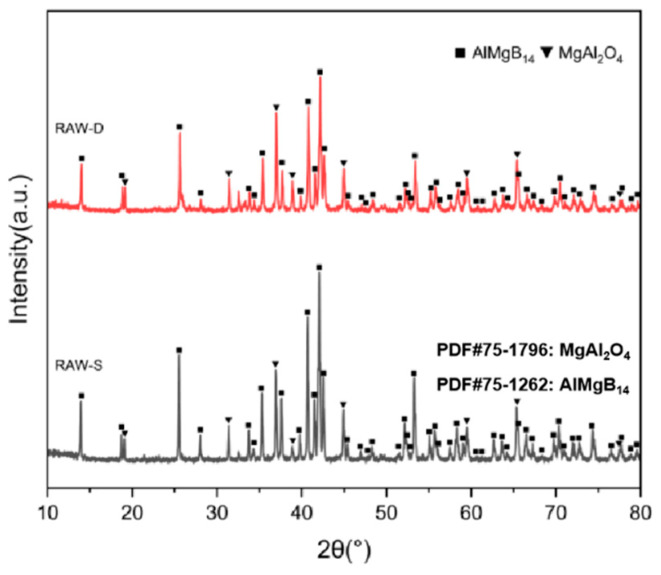
XRD patterns of RAW-S and RAW-D.

**Figure 7:**
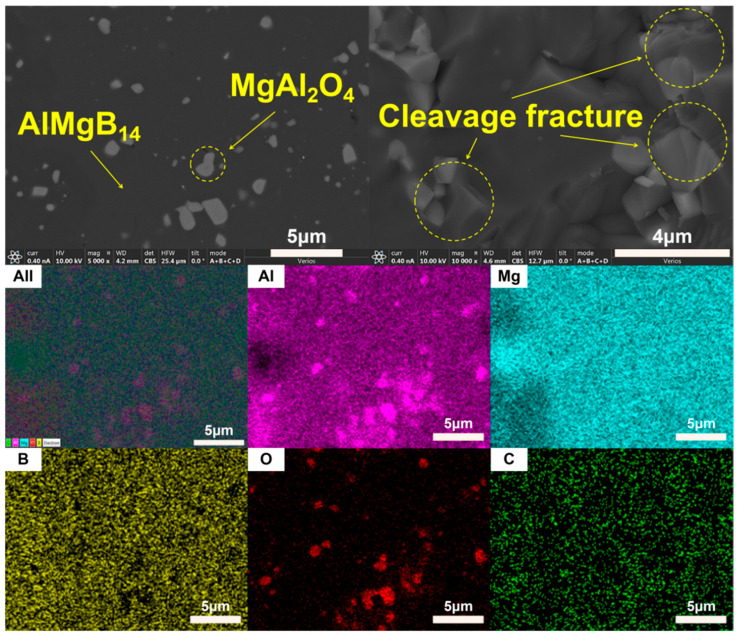
SEM images and EDS results of RAW-S.

**Figure 8:**
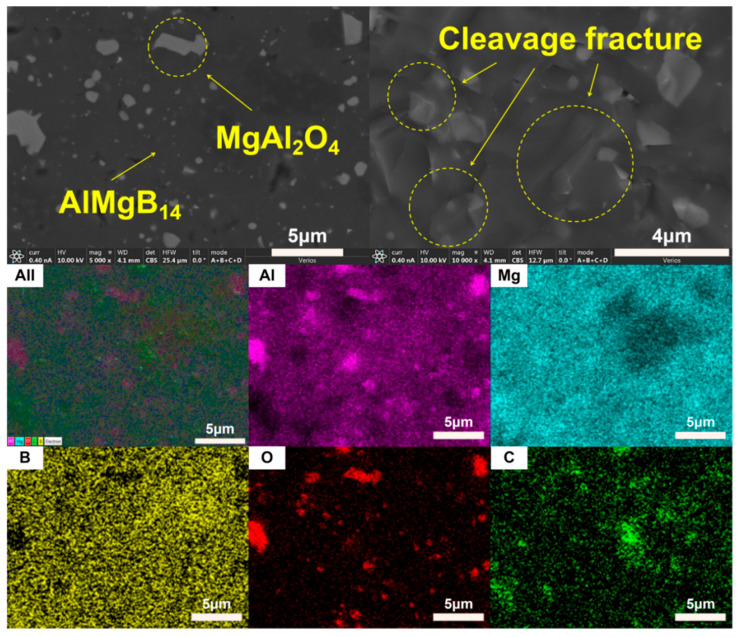
SEM images and EDS results of RAW-D.

The XRD patterns of the bulk ceramic materials obtained from the sintering of the raw powder can be seen in Figure 6. It is evident that, regardless of whether the mixed powder has undergone a second round of ball milling, the main phase compositions of the sintered bulk materials are AlMgB_14_ and MgAl_2_O_4_. The SEM images of the polished and fracture surfaces of the bulk materials are shown in Figure 7 and Figure 8. Combining the results of the surface EDS analysis, it can be determined that the darker contrast region is the ceramic matrix phase of AlMgB_14_, while the brighter contrast parts are the oxide impurity phase of MgAl_2_O_4_. It is easy to observe that the MgAl_2_O_4_ phase is distributed throughout all areas of the ceramic matrix, with a relatively uniform distribution. Moreover, the bulk materials sintered from the powder that underwent a second round ball milling process exhibit more MgAl_2_O_4_ phase distribution. This is not difficult to understand, as the powder preparation process did not involve additional protection, allowing the powder to be in contact with oxygen for a longer time, which intensified oxidation and led to an increase in the oxide phase in the sintered product. Also, based on the diffraction peaks of the matrix phase, it can be seen from the XRD patterns that the relative intensity of the diffraction peaks of the oxide phase in RAW-D was slightly higher than that in RAW-S. There were a certain number of pores in the ceramic material, which could possibly have been caused by the volatilization loss of substances such as B_2_O_3_ during the high-temperature sintering process [50,51,52,53]. Further research is needed to determine the form mechanism of pores.

The SEM images of fracture surface shown in Figure 7 and Figure 8 reveal the fracture behavior characteristics of the sintered ceramic bulk material. Regardless of whether the powder underwent a second round of ball milling treatment, the sintered ceramic bulk material exhibited typical brittle fracture characteristics. Microscopic analysis indicates that the fracture mode mainly presented as a transgranular fracture. Additionally, typical cleavage fracture characteristics can be identified in the images.

#### 3.2.2. The Effect of Single-Component Additive on the Phase Composition and Microstructure of AlMgB_14_ Material

**Figure 9:**
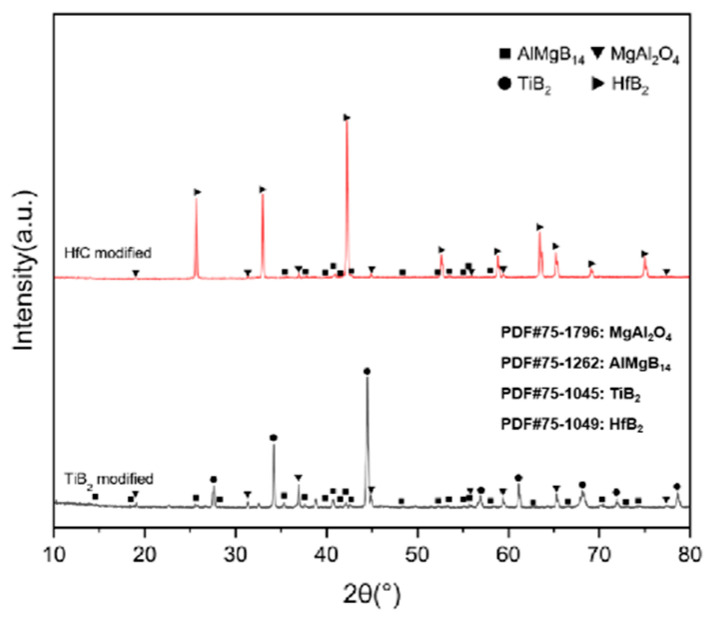
XRD patterns of the second type of sample.

For bulk materials prepared using only one additive, the XRD patterns are shown in Figure 9. In the case of preparing ceramic composites using TiB_2_, the main phase composition of the sintered bulk material is AlMgB_14_, TiB_2_, and MgAl_2_O_4_.

**Figure 10:**
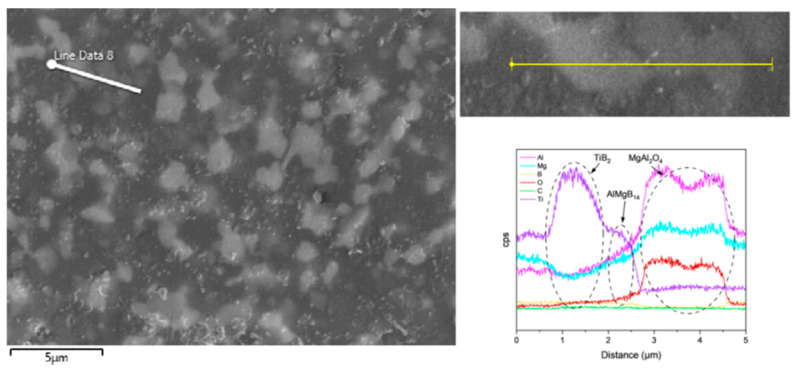
Line scan result of the TiB_2_ added sample.

**Figure 11:**
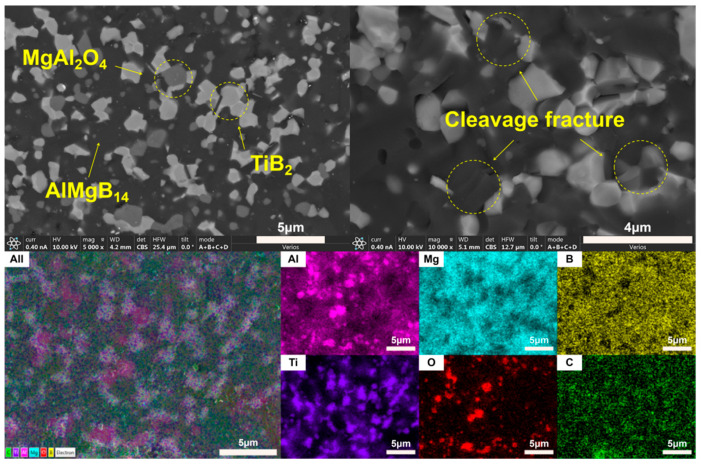
SEM images and EDS results of T.

The addition of TiB_2_ did not lead to the formation of any detectable new phases. Combining the SEM images and EDS results (Figure 10 and Figure 11), it can be observed that the TiB_2_ particles are relatively uniformly distributed in the ceramic matrix, and no significant diffusion layer was detected. The size of the TiB_2_ phase is approximately 2–3 μm.

**Figure 12:**
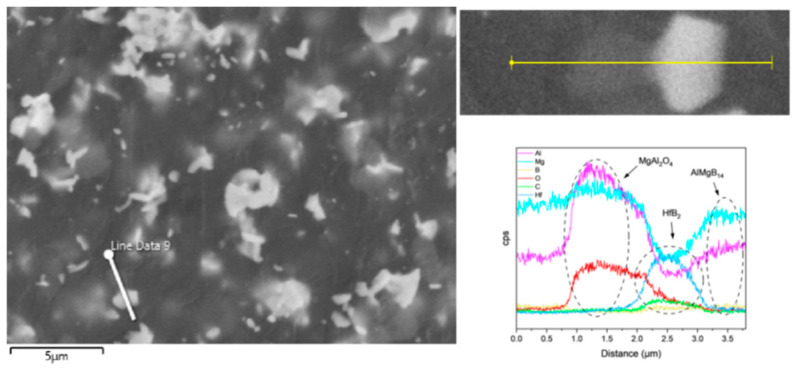
Line scan result of the HfC added sample.

**Figure 13:**
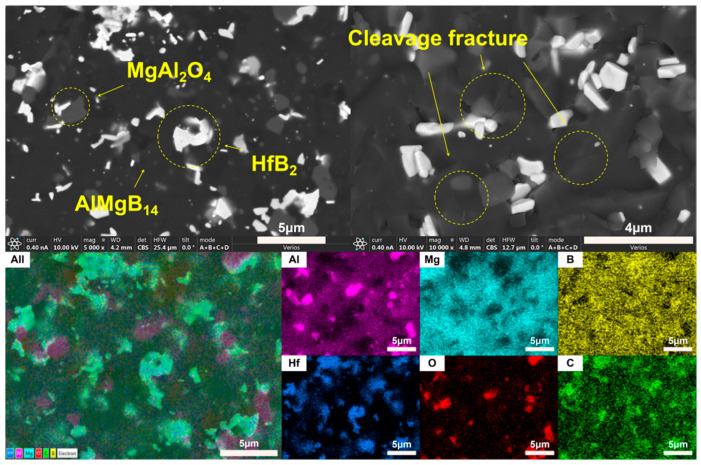
SEM images and EDS results of H.

In the ceramic composite material with HfC added, the main phase compositions (see Figure 9) were AlMgB_14_, HfB_2_, and MgAl_2_O_4_. After adding HfC, a new phase of HfB_2_ was formed, and the diffraction peak of the additive HfC itself could no longer be detected by XRD characterization. In the region of 2θ = 35–45°, extremely weak diffraction peaks of Al-Mg alloy and Al_3_B_48_C_2_, as well as some low-boron compounds such as the weak diffraction peak of AlB_12_, can be detected. Al_3_B_48_C_2_ is an isomer (β-AlB_12_) [54] of the decomposition product AlB_12_ of the ceramic matrix AlMgB_14_. Further research is needed to confirm the existence of these substances. According to the line scan results (see Figure 12), the variation of Hf element requires a relatively long distance to reach the peak concentration. There may be a reaction layer between the second phase and the ceramic matrix. The size of the HfB_2_ phase was about 1–2 μm, and the shape of this second phase was irregular. Agglomeration of some second phase particles can be observed (see Figure 13), and large second phase particle clusters could reach over 6 μm.

**Figure 14:**
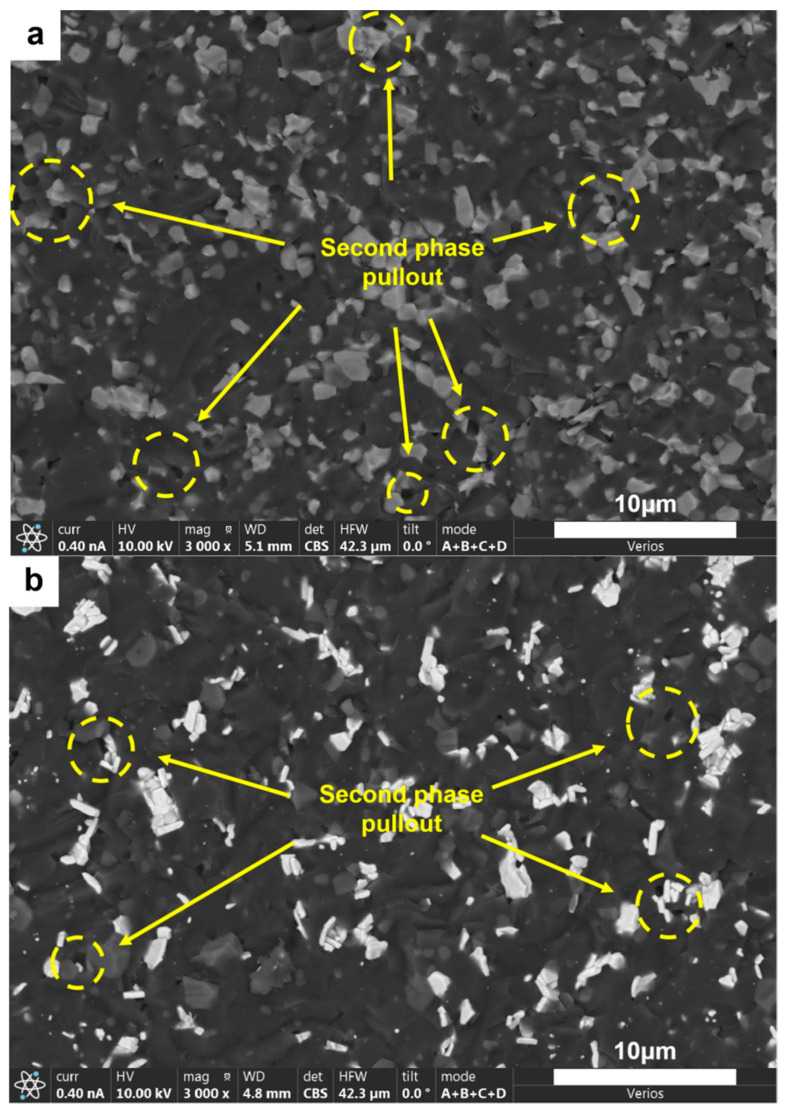
SEM image of the fracture surface of the second type of sample: (**a**) T and (**b**) H.

SEM images (Figure 11 and Figure 13) indicate that the fracture surfaces of the AlMgB_14_ ceramic composite material were all brittle fractures, and on the fracture surfaces, obvious transgranular fracture morphological features and typical cleavage fracture characteristics can be seen. In the ceramic composite material with TiB_2_ added, the phenomenon of second-phase particle pullout (see Figure 14a) in certain areas suggests that TiB_2_ had not formed a strong chemical bond with the AlMgB_14_ ceramic matrix, which has a positive effect on the improvement of material toughness and other properties. The HfC added sample exhibited different strengthening characteristics, with the observed distribution of slender columnar crystals, and a significant reduction in the phenomenon of second-phase particle pullout (see Figure 14b). This morphological transition may originate from the interfacial reaction between HfC and the matrix during the sintering process, forming a reaction layer. The presence of the reaction layer enhances the interfacial bonding strength, making crack propagation consume more energy to pull out the second phase, thus strengthening the toughening effect. Further research is still needed to confirm the validity of this hypothesis.

#### 3.2.3. The Effect of Dual-Component Synergistic Composite Strategy on the Phase Composition and Microstructure of AlMgB_14_ Material

**Figure 15:**
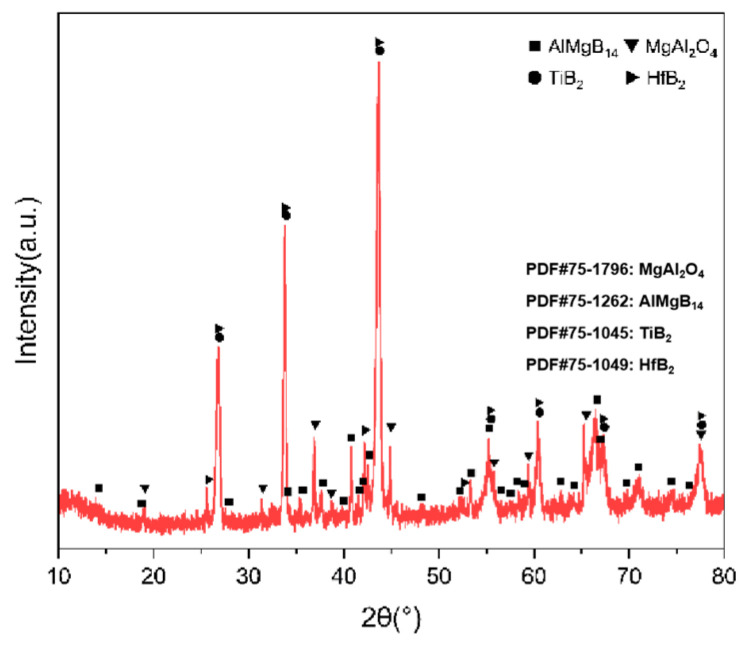
XRD pattern of TH.

For the bulk samples with two additives added, the XRD results are shown in Figure 15, and the main phase compositions are listed in Table 3.

**Figure 16:**
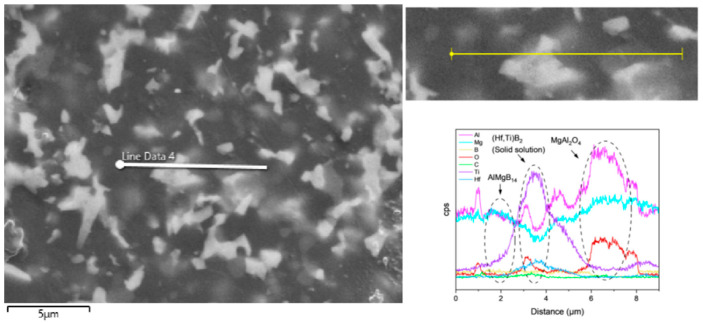
Line scan result of TH.

**Figure 17:**
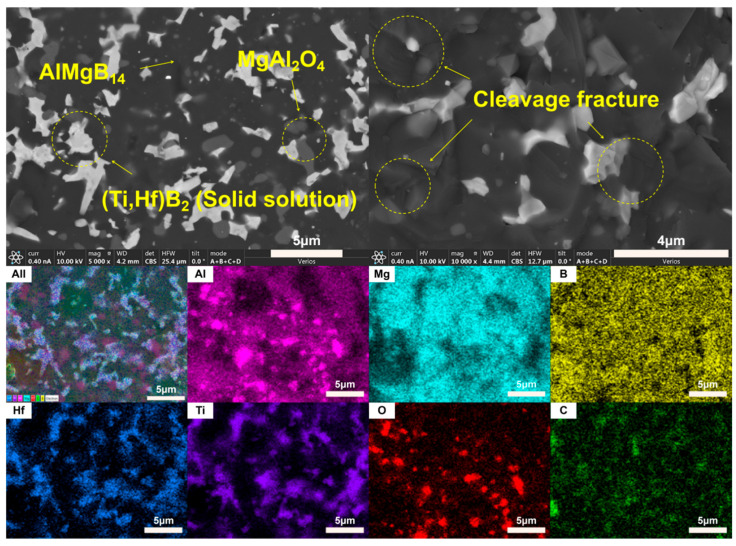
SEM images and EDS result of TH.

Based on the XRD analysis results, the main phase composition of the TiB_2_-HfC synergistic composite bulk material was AlMgB_14_, (Ti, Hf)B_2_, and MgAl_2_O_4_. Notably, the second phase in the sample exhibits a behavior that is distinctly different from the previously discussed samples. SEM images and EDS results (see Figure 16 and Figure 17) show that the distribution trends of Hf and Ti are the same. This indicates that a solid solution likely formed in the ceramic. The size and distribution pattern of the second phase remain identical to those of the sample containing only HfC, featuring columnar crystals. However, the morphology of the second phase differed from that observed in the sample with TiB_2_ added. By analyzing the XRD pattern (Figure 15), it is evident that the boride diffraction peaks in the TiB_2_-HfC added sample shift towards the low-angle region compared to pure TiB_2_. For instance, the diffraction peak values corresponding to the (001), (100), and (101) crystal planes shifted by approximately 0.76, 0.35, and 0.76 degrees towards the low angle, respectively. Combining the line scan results (Figure 11), it can be inferred that Hf had solid-solved into the TiB_2_ phase. The larger atomic size of Hf leads to an increase in the lattice constant of (Ti, Hf)B_2_ after solid-solution, which manifested as a shift in the related XRD diffraction peaks, confirming the previous hypothesis about the formation of the solid solution second phase. The formation of a solid solution in the sample with TiB_2_ and HfC added can be attributed to the HfB_2_ produced during the sintering process, which had the same crystal structure as TiB_2_ and similar lattice parameters [55,56,57,58]. Additionally, discussions by other researchers [53,59] also corroborate the rationality of the (Ti, Hf)B_2_ solid-solution formation in this work.

Observing the SEM image of the material fracture surface (Figure 17), it is evident that the bulk material prepared by adding two additives still exhibited a brittle failure mode. Distinct transgranular fracture morphological features and cleavage fracture characteristics can be observed, without a significant amount of second-phase pull-out phenomenon. Additionally, the sintered ceramic bulk appears relatively dense, with no significant pores observable. In the dual-component additive added sample, a series of complex interactions between the matrix and additives result in the formation of grains resembling long columnar and branched shapes, creating effects similar to pinning and whisker reinforcement, thereby affecting the material’s properties.

### 3.3. Properties of AlMgB_14_ Ceramic Bulk Material

Table 4 displays the fundamental properties of sintered ceramic materials, including hardness, fracture toughness, bending strength, and density. The results indicate that whether the raw powder (RAW) underwent a second round of ball milling had no significant impact on the basic properties of the bulk material. Additionally, the density of samples sintered from powders that had undergone a second round of ball milling was slightly higher than that of the sample without a second round of ball milling. Both the samples had a density higher then pure AlMgB_14_. This phenomenon can be attributed to the formation of MgAl_2_O_4_ phase during the sintering process, as the density of MgAl_2_O_4_ is higher than that of AlMgB_14_, leading to an overall increase in density for the sample with higher oxide content. Although the formation of MgAl_2_O_4_ is typically considered to weaken the material’s hardness, in this study, the samples that underwent a second round of ball milling did not exhibit a significant decrease in hardness, despite having a higher oxide phase content. We can observe that the size of the oxide particles could be quite small, with some being in the nanoscale. This anomalous behavior may be attributed to the strengthening effect of the small-sized oxide phase, which to some extent offsets the adverse effect of oxide on hardness. These results are consistent with the hypothesis proposed by Lewis et al. [5].

In terms of composite, the addition of HfC did not significantly enhance the hardness of the material. However, the introduction of TiB_2_ or the combination of TiB_2_ and HfC markedly increased the material’s hardness, reaching up to 25.3 GPa. Regarding fracture toughness, all samples with additives showed improvements to varying degrees. This enhancement can be attributed to the presence of a secondary phase in the ceramic matrix, which features columnar and branched structures. These structures effectively improve the material’s fracture toughness by pinning and hindering the propagation of cracks. Notably, samples composite with the dual-component TiB_2_-HfC additive exhibited particularly outstanding performance in fracture toughness, with an average value reaching 6.9 MPa m^1/2^, representing an increase of over 100% compared to the samples without additive.

**Figure 18:**
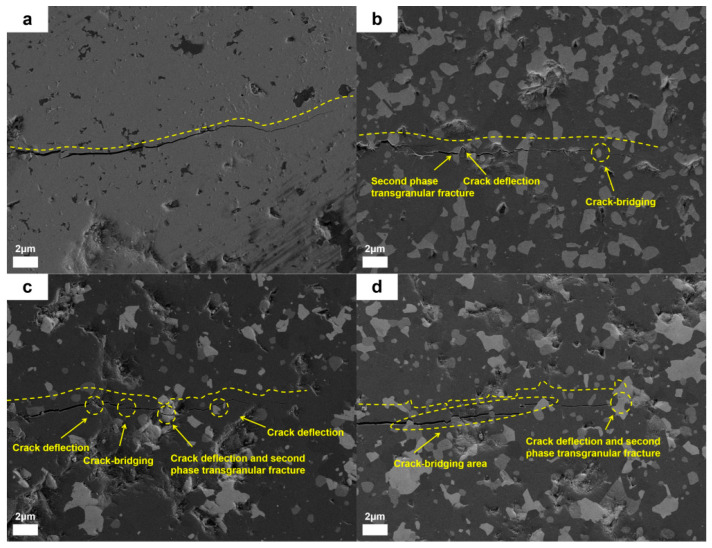
Crack propagation in ceramic bulk materials: (**a**) RAW-D, (**b**) T, (**c**) H, (**d**) TH.

As shown in Figure 18, the microscopic morphology analysis of the crack propagation path in bulk materials revealed that the samples without additive exhibited long, straight cracks with unobstructed propagation, whereas the modified samples all displayed significant toughening characteristics. In the dual-component synergistic composite system, the cracks showed a short-range twisted morphology accompanied by a typical bridging effect. The second phase formed a stress barrier by bridging the two sides of the crack, effectively absorbing the propagation energy and inhibiting crack extension. Notably, in the synergistic composite samples, the second phase not only triggered crack path deflection but also directly terminated the crack tip propagation through pinning action. This multi-scale synergistic mechanism has become the key factor in enhancing the material’s fracture toughness. These results indicate that the comprehensive mechanical properties of ceramic materials can be effectively optimized by rationally designing and introducing appropriate secondary phase additives.

Based on the demand for material lightweighting, the correlation between density and comprehensive performance of different composite systems was further evaluated. Experimental data shows that the density of the TiB_2_ single-component modification system was the lowest, at 2.99 g/cm^3^, while the density of the HfC single-component modification system was the highest, at 3.45 g/cm^3^. The density of the TiB_2_-HfC dual-component synergistic composite system was 3.22 g/cm^3^. Considering the mechanical performance of the material systems comprehensively, the TiB_2_-HfC synergistic composite system achieved a successful optimization of hardness, fracture toughness, and bending strength while maintaining a relatively low density, demonstrating the most outstanding comprehensive performance.

## 4. Conclusions

This study proposed a scheme for preparing AlMgB_14_ ceramic materials with dual-component composite, and successfully prepared AlMgB_14_-based ceramic composites through a two-step heat treatment method.

1. Through systematic analysis of the phase composition and morphology of ceramic powders, the influence of heat treatment temperature on the phase composition was clarified. The study found that the intermediate product Al_0.5_Mg_0.5_B_2_ begins to convert into the target product AlMgB_14_ in large quantities at 1100 °C, and the conversion tends to be complete at 1150 °C. The reaction paths of the product and by-products were also revealed.
2. After modification with a 30 wt.% (TiB_2_ and HfC in a 1:1 mass ratio) addition of a dual-component additive, the overall performance of the material is significantly optimized. The Vickers hardness is maintained at a level of 25.3 GPa, while the fracture toughness is improved to 6.9 MPa m^1/2^, which is an increase of over 100% compared to the samples without additive. The bending strength reaches 615 MPa, and the density is controlled at 3.22 g/cm^3^. It maintains a high level of fracture toughness, hardness, and bending strength while also having a low density.
3. Through the analysis of the crack propagation behavior of the dual-component composite AlMgB_14_ ceramic material, it was found that its microscopic fracture mechanism exhibits significant synergistic toughening characteristics, with an obvious crack deflection phenomenon. At the same time, typical crack bridging areas can be observed, which can absorb a considerable amount of energy when cracks occur, thereby inhibiting the propagation of cracks. Furthermore, the pull-out effect of the second phase further suppresses the unstable propagation of cracks.

This study provides an efficient method for the preparation of AlMgB_14_ ceramics and their composites and achieves synergistic enhancement of material properties through composite strategies, offering new insights for the design and preparation of high-performance ceramic materials.

## Figures and Tables

**Table 1:** Manufacturers and specifications of raw materials.

Powder	Manufacturer	Particle Size	Purity
Al	Shanghai Xiangtian Nanomaterials Co., Ltd., Shanghai, China	5 μm	99.9%
Mg	Shanghai Xiangtian Nanomaterials Co., Ltd., Shanghai, China	15 μm	99.9%
B	Qinhuangdao Eno High-Tech Material Development Co., Ltd., Qinhuangdao, China	0.5–2 μm	95.0%
TiB_2_	Qinhuangdao Eno High-Tech Material Development Co., Ltd., Qinhuangdao, China	0.5–1 μm	99.5%
HfC	Qinhuangdao Eno High-Tech Material Development Co., Ltd., Qinhuangdao, China	1–3 μm	99.5%

**Table 2:** Phase composition of powders.

Sample	Main Phases
RAW	Al, Mg, B (amorphous)
950 °C	Al_0.5_Mg_0.5_B_2_, MgAl_2_O_4_, B (amorphous)
1000 °C	Al_0.5_Mg_0.5_B_2_, MgAl_2_O_4_, B (amorphous)
1050 °C	Al_0.5_Mg_0.5_B_2_, AlMgB_14_, MgAl_2_O_4_
1100 °C	AlMgB_142_, Al_0.5_Mg_0.5_B, MgAl_2_O_4_
1150 °C	AlMgB_14_, MgAl_2_O_4_
1200 °C	AlMgB_14_, MgAl_2_O_4_

**Table 3:** Phase composition of sintered ceramic materials.

Sample	Main Phases
RAW-Single (RAW-S)	AlMgB_14_, MgAl_2_O_4_
RAW-Double (RAW-D)	AlMgB_14_, MgAl_2_O_4_
TiB_2_ Modified (T)	AlMgB_14_, TiB_2_, MgAl_2_O_4_
HfC Modified (H)	AlMgB_14_, HfB_2_, MgAl_2_O_4_
TiB_2_-HfC Modified (TH)	AlMgB_14_, (Ti, Hf)B_2_, MgAl_2_O_4_

**Table 4:** The main performance indicators of ceramic bulk materials.

Sample	Density (g/cm^3^)	Hardness (HV1, GPa)	Fracture Toughness (MPa·m^1/2^)	Bending Strength (MPa)
RAW-S	2.61	23.2 ± 0.4	3.16 ± 0.17	507 ± 58
RAW-D	2.64	23.6 ± 0.4	3.29 ± 0.10	489 ± 40
T	2.99	25.3 ± 0.5	5.91 ± 0.52	646 ± 46
H	3.43	23.3 ± 0.2	4.97 ± 0.48	559 ± 55
TH	3.22	25.3 ± 0.9	6.94 ± 0.49	615 ± 118

## Data Availability

The original data related to this research can be requested for reasonable use at any time by sending an email to the author: suntianxing22@mails.ucas.ac.cn.
